# Complete genome sequence of a novel potyvirus infecting *Miscanthus sinensis* (silver grass)

**DOI:** 10.1007/s00705-022-05445-3

**Published:** 2022-05-17

**Authors:** Zacharie Leblanc, Marie-Emilie Gauthier, Ruvini Lelwala, Candace Elliott, Cassie McMaster, Robin Eichner, Kevin Davis, Lia Liefting, Jeremy Thompson, Adrian Dinsdale, Mark Whattam, Julie Pattemore, Roberto A. Barrero

**Affiliations:** 1 eResearch Office, Queensland University of Technology, Brisbane, QLD 4000 Australia; 2 Science and Surveillance Group, Department of Agriculture, Water and the Environment, Post Entry Quarantine, Mickleham, VIC 3064 Australia; 3 Department of Agriculture, Water and the Environment, Plant Innovation Centre @ Post Entry Quarantine, Mickleham, VIC 3064 Australia; 4 Department of Agriculture, Water and the Environment, Australian Government, Canberra City, ACT 2601 Australia; 5 Plant Health and Environment Laboratory, Ministry for Primary Industries, P.O. Box 2095, Auckland, 1140 New Zealand

## Abstract

Here, we describe the full-length genome sequence of a novel potyvirus, tentatively named “Miscanthus sinensis mosaic virus” (MsiMV), isolated from *Miscanthus sinensis* (silver grass) held in a post-entry quarantine facility after being imported into Western Australia, Australia. The MsiMV genome is 9604 nucleotides (nt) in length, encoding a 3071-amino-acid (aa) polyprotein with conserved sequence motifs. The MsiMV genome is most closely related to that of sorghum mosaic virus (SrMV), with 74% nt and 78.5% aa sequence identity to the SrMV polyprotein region. Phylogenetic analysis based on the polyprotein grouped MsiMV with SrMV, sugarcane mosaic virus (SCMV), and maize dwarf mosaic virus (MDMV). This is the first report of a novel monopartite ssRNA virus in *Miscanthus sinensis* related to members of the genus *Potyvirus* in the family *Potyviridae*.

*Miscanthus sinensis,* commonly known as silver grass, is a member of the family Poaceae and is native to eastern Asia, where it is a keystone species in grasslands. In addition to ornamental applications, *M. sinensis* has been investigated for use as a biofuel crop due to its high biomass yield. Miscanthus has been found to be an alternate host for viruses infecting plants of the family Poaceae, including switchgrass mosaic virus and barley yellow dwarf virus [1].

Potyviruses represent the largest and most economically damaging genus of known plant RNA viruses [2]. Potyviruses are aphid-borne, infecting both monocotyledonous and eudicotyledonous angiosperms [3]. In response to viral infection, plants can disrupt viral transcripts via RNA interference using Dicer-like endonucleases [4]. Infections by potyviruses typically result in silencing responses, predominantly using 21- and 22-nt small RNAs [5].

All potyviruses have a monopartite genome containing a long open reading frame (ORF) [6], which encodes a polyprotein that is proteolytically processed by three viral proteases into 10 functional proteins: P1, HC-Pro, P3, 6K1, cylindrical inclusion (CI) protein, 6K2, viral genome-linked protein (VPg), NIa-Pro, nuclear inclusion b (NIb; also known as RNA-dependent RNA polymerase), and capsid protein (CP) [7, 8].

A *Miscanthus sinensis* cultivar “Morning Light” plant imported into Western Australia from the USA in 1985 was transferred to the Plant Quarantine Station, Rydalmere, New South Wales, for virus screening, as per new import conditions for clonal grasses at the time [9]. Double-stranded RNA extracted from 38 g of mature leaf tissue electrophoresed in a 0.75% agarose gel revealed bands suspected to be of viral RNA, and subsequent mechanical inoculation of ground grass tissue onto plants of *Zea mays* cv. Supagold resulted in mosaic symptoms [10]. The infected Morning Light cultivar was never released from post-entry quarantine and was maintained as a positive control for biological indexing (Fig. 1A). Sap from the infected plant contained filamentous particles approximately 600 nm in length (Fig. 1B), further confirming the presence of a potyviral pathogen.

**Fig. 1:**
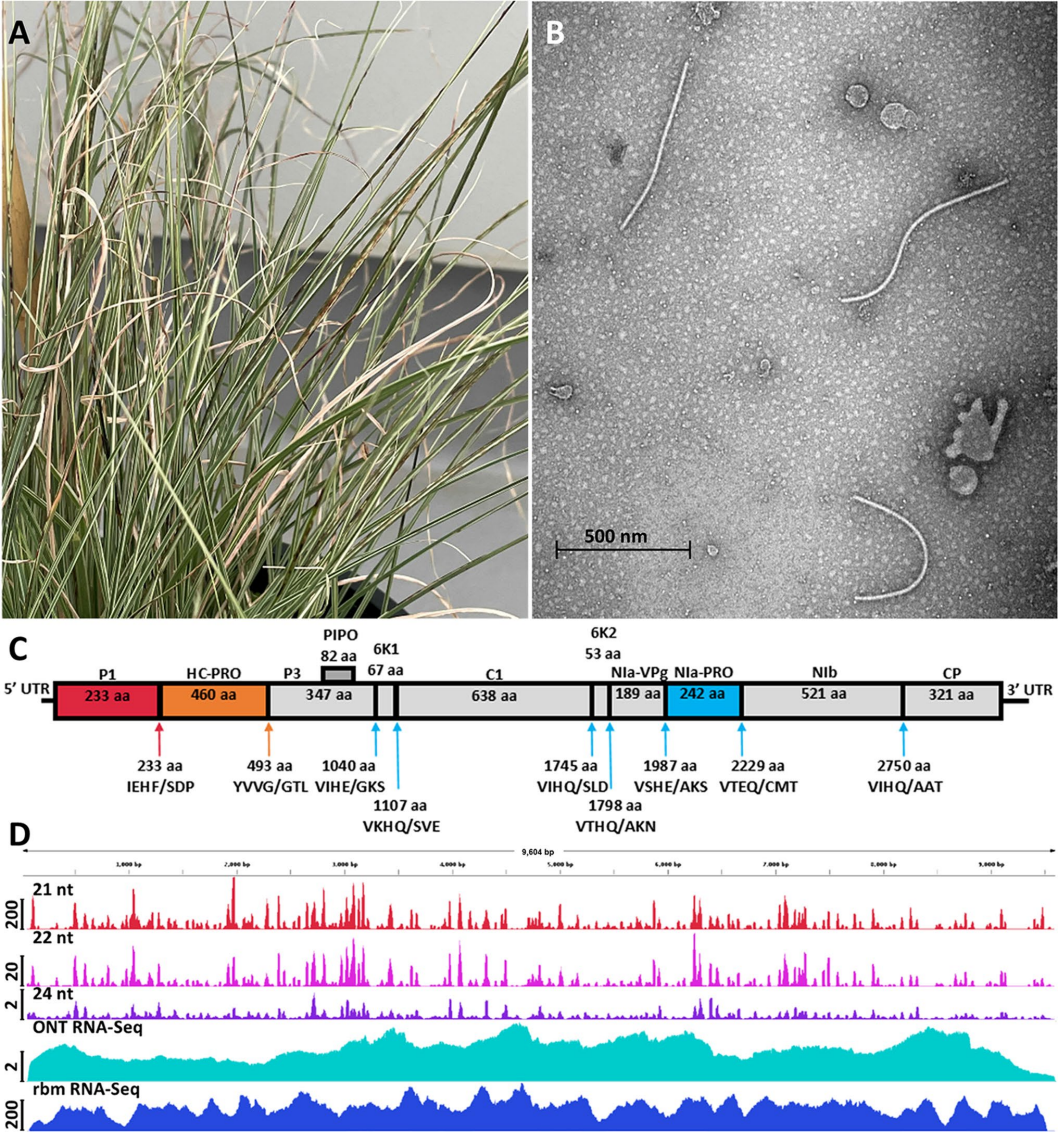
(A) A *Miscanthus* plant infected with MsiMV, showing leaf mottling symptoms. (B) Morphology of MsiMV virions viewed by transmission electron microscopy. Samples were prepared using a modified ‘quick dip’ method [14]. Freshly cut leaf pieces (2 × 2 mm) were placed into 2% (w/v) phosphotungstic acid stain (pH 7.07) and adsorbed onto a carbon-coated mesh grid (formvar carbon film 400 mesh, copper). Grids were dried at 37 °C for at least 30 min then examined at 26,000x magnification using a Tecnai G2 Spirit transmission electron microscope. (C) Schematic representation of the genome organization of MsiMV. The 5′ (140 nt) and 3′ (251 nt) untranslated regions are represented by solid horizontal bars, while ORFs are depicted as open boxes. Putative sites of polyprotein cleavage mediated by P1, HC-Pro, and NIa-Pro are indicated by red, orange, and blue arrows, respectively. Protein domain sizes in aa are indicated below the protein name. (D) Alignment of small RNAs, ONT, and Illumina ribominus RNA-seq sequences with the MsiMV genome sequence. Scale bars on the *y*-axis show the total number of mapped reads (x1000).

Collection of RNA samples and *in silico* assembly of 21- to 22-nt small RNA sequences were performed as described previously [11], yielding a single 9604-nt sequence containing a single long ORF from nt 141 to 9353 and flanking non-coding sequences, including a polyA tail at the 3’ end, which is typical of potyviruses. Mapping of small RNA reads onto the MsiMV genome sequence, allowing no mismatches, resulted in 11,370,140 aligned reads (~25Kx coverage) of which 89.2%, 7.6%, and 0.3% were 21 nt, 22 nt, and 24 nt long, respectively. The high proportion of mapped 21-nt reads is consistent with them being derived from the antiviral silencing response [5].

The completeness of the small-RNA-based genome assembly was confirmed by mapping of both Illumina and long-read Oxford Nanopore Technology (ONT reads > 500 bp) ribosomal-depleted RNA-seq data (Fig.1D). Overall, 7,154,030 Illumina (~74.5Kx coverage) and 21,495 ONT (2140x coverage) reads were mapped onto the MsiMV genome using bowtie [12] and minimap2 [13], respectively. Identical assembled MsiMV genome sequences were obtained using all three technologies. Raw data are available under BioProject PRJNA752836.

The qualifiers accepted by the International Committee on Taxonomy of Viruses for a new species of potyvirus are < 76% nt and < 82% aa ORF sequence identity [8]. Comparison of the MsiMV polyprotein and coat protein sequences against those of SrMV (KM025045) showed that the new virus met these criteria. Additionally, the polyprotein is predicted to be cleaved into 10 subdomains corresponding to those of other potyviruses (Fig. 1C).

Phylogenetic analysis based on polyprotein sequences placed MsiMV within a clade with SrMV and MDMV, with SCMV as a sister clade (Fig. 2). The clade formation of these viruses may be reflective of the similarity of their plant hosts, which are all in the subfamily Panicoideae of the family Poaceae. The sequence similarity of these viruses could also explain overlaps in host susceptibility seen with SrMV, which can infect *Miscanthus,* corn, and sugarcane. The phylogenetic position of MsiMV, separate from SrMV, SCMV, and MDMV, further supports the classification of MsiMV as a member of a novel species in the genus *Potyvirus*.

**Fig. 2:**
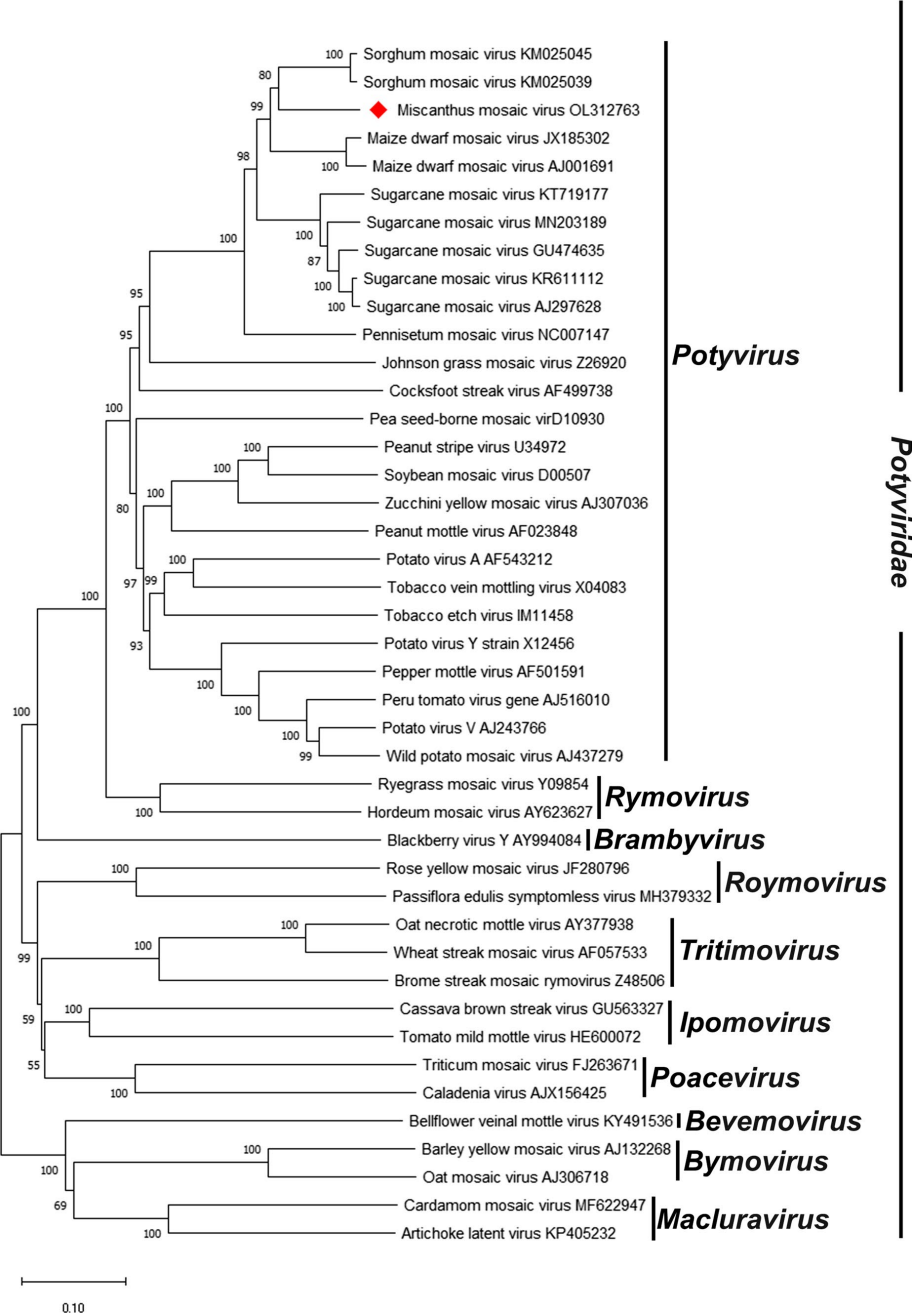
Neighbor-joining tree of polyprotein aa sequences of MsiMV and selected members of the family *Potyviridae*. Bootstrap analysis was applied, using 1000 bootstrap replicates in MEGA X [15]. Percent bootstrap values are shown at each node.
